# The TMEM63B Channel Facilitates Intestinal Motility and Enhances Proliferation of Intestinal Stem Cells

**DOI:** 10.3390/cells13211784

**Published:** 2024-10-28

**Authors:** Jing-Jing Tu, Yan-Yu Zang, Yun Stone Shi, Xiao-Yu Teng

**Affiliations:** 1 Model Animal Research Center, Jiangsu Key Laboratory of Molecular Medicine, Medical School, Nanjing University, Nanjing 210093, China; tujj@smail.nju.edu.cn (J.-J.T.); dg21350022@smail.nju.edu.cn (Y.-Y.Z.); 2 Guangdong Institute of Intelligence Science and Technology, Hengqin, Zhuhai 519031, China

**Keywords:** TMEM63B, intestinal stem cells (ISCs), gastrointestinal motility and digestion, colitis, ISCs proliferation

## Abstract

The intestines are in a constant state of motion and self-renewal. The mechanical breakdown of food facilitates intestinal movement and aids digestion. It is believed that mechanical stimulation, triggered by changes in osmotic pressure within the intestines, plays a crucial role in regulating gastrointestinal motility. While TRPs and PIEZO1/2 have been identified as mechanosensitive ion channels involved in this process, there still exist numerous unidentified channels with similar properties. In this study, we demonstrate that the TMEM63B expressed in intestinal stem cells contributes to the regulation of intestinal motility and digestion. The deletion of TMEM63B in intestinal stem cells not only decelerates intestinal motility and impairs digestion but also attenuates the proliferation of intestinal stem cells and exacerbates DSS-induced colitis in mice. Collectively, our findings unveil the pivotal role of TMEM63B in governing optimal digestive function and modulating intestinal motility.

## 1. Introduction

The small intestine, the most rapidly renewing tissue in mammals, is sustained by intestinal stem cells (ISCs) that facilitate intestinal development, self-renewal, and regeneration [1]. ISCs, also known as crypt base columnar (CBC) cells labeled by the LGR5 protein, are situated at the base of the intestinal crypts and intercalated between terminally differentiated secretory Paneth cells [2,3,4]. ISCs serve as the primary source of various types of intestinal cells for self-renewal, replenishment, and repair in response to stress injuries [5,6,7]. On average every 24 h, ISCs undergo differentiation and generate rapidly proliferating cells for self-renewal purposes. The invaginations of intestinal crypts give rise to ISCs which continuously proliferate and generate transit amplifying (TA) cells [8]. Moreover, mechanical stress resulting from the expansion of proliferative ISCs at the base of the crypts induces the migration of TA cells towards the villus surface where they eventually undergo apoptosis within one week [9,10]. After several rounds of cell cycles, TA cells exit from their cell cycle progression and differentiate into specific intestinal cell types through proliferation mechanisms. These include absorptive enterocytes characterized by a highly polarized columnar morphology with a delicate luminal brush-like border; goblet cells and enteroendocrine cells distributed throughout both villi and crypts responsible for mucus secretion and hormone production, respectively; tuft cells located along the entire length of the crypt–villus axis involved in sensing luminal contents; Paneth cells closely associated with small ISCs secreting Wnt signaling molecules regulating ISC proliferation and differentiation [11,12,13]; microfolded (M) cells playing a role in mucosal immunity acting as sentinels for luminal antigens [1].

The intestinal epithelium is composed of a continuous monolayer of tightly connected intestinal epithelial cells, forming a protective barrier against external damage [14]. The intestinal epithelium exhibits rapid self-renewal ability, heavily relying on the continuous proliferation and differentiation of ISCs for the production of intestinal epithelial cells [3,15]. Notably, intestinal epithelial injury can lead to further inflammatory responses such as inflammatory bowel disease (IBD) [14], including Crohn’s disease (CD) and ulcerative colitis, which are chronic and complex diseases characterized by long-term pathogenic inflammation and damage to the intestinal epithelium [16]. Normally, the mucosal immune system within the intestines coevolves with intestinal bacteria to sustainably respond to invading pathogens [17]. In an ulcerative colitis model induced by dextran sulfate sodium (DSS) in mice, the invasion of intestinal bacteria into intestinal epithelial cells significantly enhances the inflammatory response at the site of injury within the intestines. Following acute enteritis injury, the repair phase is rapidly initiated where ISCs begin proliferating and differentiating to restore damaged epithelium.

The mechanical stimulation in the intestines originates from changes in daily diet and food digestion within the intestinal tracts [18]. Previous studies have revealed that mechanical forces play a significant role in influencing the fate of ISCs and maintaining homeostasis [19,20,21]. Recently, we and other groups have demonstrated the pivotal role of TMEM63 family proteins as mechanosensitive ion channels in various physiological processes including thirst regulation, auditory perception, the secretion of thyroid hormone and pulmonary surfactant [22,23,24,25,26,27,28,29]. In this study, we observed an abundant expression of TMEM63B in ISCs. The deletion of TMEM63B in ISCs resulted in more severe inflammation and greater colon shortening compared to the control mice after treatment with DSS. Furthermore, enteritis-induced colonic distention further disrupted the mechanical microenvironment within the intestines. However, the conditional knockout (cKO) of TMEM63B specifically in ISCs prevented the rapid proliferation and repair of a damaged epithelium when colitis occurred, ultimately leading to more severe colitis symptoms. Overall, our findings demonstrated that TMEM63B functions as a critical mechanosensitive ion channel involved in regulating the proliferation of ISCs, thereby impacting intestinal motility and digestion.

## 2. Materials and Methods

### 2.1. Animals

The animal experiments were conducted in accordance with the protocols approved by the Institutional Animal Care and Use Committee at the Model Animal Research Center (MARC) of Nanjing University, Nanjing, China, following guidelines set by Association for Assessment and Accreditation of Laboratory Animal Care (AAALAC). The mice were housed under specific-pathogen-free (SPF) conditions with a 12 h light–dark cycle and provided free access to water and food. Male and female mice aged between 8 and 16 weeks were used in this study. Detailed information is provided in the figures and legends. The mice with a specific deletion of TMEM63B in ISCs were generated by crossing *Tmem63b^HA-fl/HA-fl-^* mice (GemPharmatech Co., Ltd., Nanjing, China) with *LGR5-Cre* mice (Jackson Laboratories) (*LGR5-Cre*; *Tmem63b^HA-fl/HA-fl^*). PCR analysis of toe genomic DNA was performed to distinguish the different genotypes of all mouse strains (Novoprotein, Shanghai, China, DM005). Mice genotyping (GenScript, Nanjing, China, E00019) was performed using the following primers: LGR5-Cre common: 5′-CTGCTCTCTGCTCCCAGTCT-3′; LGR5-Cre WT: 5′-ATACCCCATCCCTTTTGAGC-3′; LGR5-Cre mutant: 5′-GAACTTCAGGGTCAGCTTGC-3′. *LGR5-Cre*; *Rosa26-LSL-tdTomato*; *Tmem63b^HA-fl/+^* mice were generated by mating *LGR5-Cre*; *Tmem63b^HA-fl/HA-fl^* mice with *Rosa26-LSL-tdTomato* mice [25].

### 2.2. Histology and Immunohistochemistry

Mice were anesthetized using isoflurane inhalation and subsequently euthanized by cervical dislocation. The intestines were promptly excised, washed with PBS (Absin, Shanghai, China, abs962), immersed with 4% paraformaldehyde (PFA), and fixed overnight at 4 °C. Next, the tissues were dehydrated using isopropanol and ethanol, embedded in paraffin, and sliced into 5 μm sections for morphological observations through H&E staining (Solarbio, Beijing, China, G1121). Immunohistochemical analysis was performed on frozen sections. The intestinal tissues were fixed in 4% PFA at room temperature (RT) for 3 h followed by dehydration in 30% sucrose solution at 4 °C for 2 days. The tissues were then embedded in optimum cutting temperature (O.C.T.) embedding medium (SAKURA, Tokyo, Japan, 4583), sliced into 10 μm sections (RWD, Shenzhen, China, FS800A), and stored at −80 °C for further use. The slices were washed three times with PBS, permeabilized with a solution of 0.3% Triton X-100 in PBS for 15 min, and blocked for 120 min using blocking buffer (PBS containing 0.3% Triton X-100 and 10% normal sheep serum) [30,31]. They were subsequently incubated overnight at 4 °C with primary antibodies diluted in blocking buffer. The primary antibodies used were rabbit anti-HA (1:250, Cell Signaling Technology, Danvers, MA, USA, 3724), rabbit anti-capase3 (1:500, Cell Signaling Technology, 9664), or mouse anti-BrdU (1:500, Abcam, Cambridge, MA, USA, ab6326). After being washed thrice with PBS, the slices were incubated at RT for 2 h with specific secondary antibodies diluted in blocking buffer. The secondary antibodies used were Alexa Fluor 488 Donkey anti-Rabbit (1:500, Invitrogen, Waltham, MA, USA, A21206), Alexa Fluor 488 Donkey anti-Mouse (1:500, Invitrogen, A32766) or Alexa Fluor 594 Donkey anti-mouse (1:500, Invitrogen, A-21203). Following three washes with PBS, the slices were counterstained with DAPI (1:1000, Keygen Biotech, Nanjing, China, KGE2505-10) for 15 min at RT. Confocal images were acquired using confocal laser-scanning microscopes (Zeiss, Jena, Germany, LSM800).

### 2.3. Stools Collection and Analysis

The mice were individually housed in iron cages with mesh at the bottom, and their feces were collected after defecation. Collection time was standardized to minimize the influence of diurnal cycle. Fresh fecal samples were immediately transferred into clean EP tubes (Jet Biofil, Guangzhou, China, CFT800015) to prevent moisture loss. Subsequently, the collected fresh feces were weighed and then dried overnight at 55 °C. The dried feces were re-weighed to determine their water content. To assess the size of the feces, they were measured for length using a vernier caliper. For colon motility measurement, mice underwent anesthesia via the inhalation of isoflurane followed by euthanasia through cervical dislocation. Colons were rapidly excised (SAINING, Suzhou, China, 4012010), and the number of retained feces was counted along with wet and dry mass measurements.

### 2.4. Food Consumption

The mice were housed in a controlled environment with a 12 h light–dark cycle and provided ad libitum access to water and food. Prior to the experiments, the mice were individually bred for three days to acclimate to their surroundings. Food consumption was determined by measuring the daily reduction in food weight over a period of 7 days, which was then averaged.

### 2.5. Sucrose Digestion

The mice were subjected to a 16 h food deprivation period. Subsequently, the mice received an oral gavage of sucrose (3 mg/g body weight). Blood samples were then collected (NEST Biotechnology, Wuxi, China, 801002) from the tail at time intervals of 0, 15, 30, 45, 60, and 120 min following glucose administration. Glucose levels in the collected blood samples were measured using a blood glucose meter (Roche, Basel, Switzerland, 1620368).

### 2.6. Whole Gastrointestinal Transit

The mice were individually placed in clean cages with access to chow pellets and water, and then gavage with the prepared carmine red paste (Sigma, St. Louis, MO, USA, C1022) of the same volume, which was maintained between 200 and 300 μL. The measurements were taken between 8:00 and 9:00 am local time. Cochineal red paste was prepared by dissolving 6% cochineal red in 0.5% 400 cP methylcellulose (Sigma, 19-2930). Feces were monitored every 15 min until the first appearance of a red fecal pellet. The time from gavage to the appearance of the first red feces is considered as the entire gastrointestinal (GI) transit time. Mice used for testing GI transit time can also be utilized for feces collection and colonic motility analysis; however, each experiment must be separated by more than 7 days.

### 2.7. In Vivo Imaging

The FITC-dextran was dissolved in PBS at a concentration of 200 μg/mL. All reagents were freshly prepared, while FITC-dextran was stored in a light-protected environment. Prior to conducting the experiment, the abdominal hair of the mice must be removed. Under ad libitum access to food and water conditions, the mice were orally administered an equal volume (200 or 300 μL) of FITC-dextran solution (Sigma, 46944) between 8 a.m. and 9 a.m., followed by individual placement into clean cages with the provision of chow pellets and water. Mice were anesthetized with isoflurane at either 1, 3, or 6 h post-gavage. The intact gastrointestinal tracts were rapidly excised at different time points and imaged using the in vivo optical imaging system (Revvity, Shanghai, China, IVIS-Lumina S5). For analysis purposes, Living Image Software (Perking Elmer, Waltham, MA, USA, version 4.7.4, 64-bit) was utilized to draw ROIs delineating the stomach and the rest of the GI tract.

### 2.8. DSS-Induced Colitis

Male littermate mice aged 8–12 weeks with a similar body weight were orally administered with autoclaved drinking water containing 3% (wt/vol) DSS (MP Biomedicals, Santa Ana, CA, USA, 02160110-CF) for a duration of 8 days to induce experimental colitis. The drinking water was replaced every third day. On the eighth day, the mice were euthanized for colon collection. Daily measurements of body weight and assessment of occult/gross blood presence were conducted.

### 2.9. Intestinal Epithelial Cells Proliferation

To assess the proliferation of small intestinal epithelial cells, we employed 5-bromo-2′-deoxyuridine (BrdU) to label the actively dividing ISCs. BrdU (Sigma, B5002) was administered intraperitoneally at a dosage of 100 mg/kg body weight. After 1 h, the mice were anesthetized with isoflurane and euthanized by cervical dislocation. Jejunum specimens were promptly collected and fixed in 4% PFA for histology and immunohistochemistry analysis. The ImageJ software (NIH, Bethesda, MD, USA, version 2.1.0) was utilized to quantify both the number of BrdU-positive cells within each intestinal crypt as well as the depth of the crypts and length of jejunal villi.

### 2.10. Statistical Analysis

All statistical analyses were performed using GraphPad Prism 8. All the data are presented as means ± SEM for the indicated number (n) of individual repeats or number of cells. The statistical tests applied are described in the figure legends. The evaluation of statistical significance was performed using either two-tailed unpaired Student’s *t*-test or two-way analysis of variance (ANOVA) with Bonferroni test for multiple comparisons. In all figures, n.s., no significance, * *p* < 0.05, ** *p* < 0.01, *** *p* < 0.001, **** *p* < 0.0001.

## 3. Results

### 3.1. TMEM63B Is Expressed in Small Intestine

**Figure 1:**
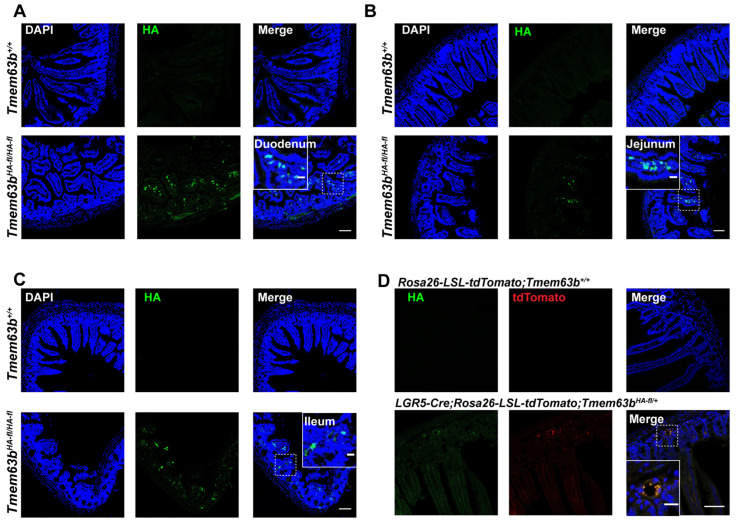
TMEM63B is expressed in the small intestine. (**A**) Representative images showing the localization of HA-TMEM63B (green) and nuclei (blue) in the duodenum. The solid frame represents an enlargement of the dotted frame. The scale bars represent 100 μm for low-magnification images and 20 μm for high-magnification images. (**B**) Representative images showing the localization of HA-TMEM63B (green) and nuclei (blue) in the jejunum. The solid frame represents an enlargement of the dotted frame. The scale bars represent 100 μm for low-magnification images and 20 μm for high-magnification images. (**C**) Representative images showing the localization of HA-TMEM63B (green) and nuclei (blue) in the ileum. The solid frame represents an enlargement of the dotted frame. The scale bars represent 100 μm for low-magnification images and 20 μm for high-magnification images. (**D**) Representative colocalization of tdTomato-positive cells expressing LGR5 marker protein (red), with HA-TMEM63B (green). The solid frame represents an enlargement of the dotted frame. The scale bars represent 200 μm for low magnification images and 50 μm for high magnification.

To investigate the expression pattern of TMEM63B in the mouse intestines, we generated a knock-in (KI) reporter mouse expressing HA-FLAG-tagged TMEM63B, with the tags strategically inserted after the N-terminal signal sequence in exon 2 [25]. Additionally, two *LoxP* segments were inserted at the first and fourth introns to facilitate the generation of cKO mice. Subsequently, we examined TMEM63B localization in the mouse intestines using an HA antibody for immunostaining. The results showed the luminal and basal expression of TMEM63B at the base of the crypts of duodenum, jejunum, and ileum epithelia (Figure 1A–C). To assess whether TMEM63B is specially expressed in ISCs, we crossed *LGR5-Cre*; *Tmem63b^HA-fl/HA-fl^* mice with *Rosa26-tdTomato* mice to generate *LGR5-Cre*; *Rosa26-tdTomato*; *Tmem63b^HA-fl/^^+^* mice. Interestingly, colocalization between HA-TMEM63B and tdTomato was observed at the crypt region (boxed area) of the small intestine (Figure 1D). Taken together, these findings suggest that TMEM63B is enriched in the small intestine, particularly within ISCs.

### 3.2. Deletion of TMEM63B in ISCs Decelerates Gastrointestinal Motility

After food intake, the digestion of food leads to an increase in intestinal contents, subsequently inducing the expansion of intestinal volume. This expansion activates mechanosensitive ion channels expressed in the intestines, such as TRPs [32,33] and PIEZO channels [19,21,34,35]. Previous study has demonstrated that TrpA1 channel is involved in regulating ISCs proliferation in *Drosophila* [36]. Additionally, it has been reported that PIEZO channel expressed on chromaffin cells of Drosophila can regulate intestinal transport [19]. In this study, we identified that TMEME63B, a mechanosensitive ion channel [22,23,25,28], is highly expressed in the gut and may play a role in regulating GI motility (Figure 1). To analyze the function of the TMEM63B channel in ISCs, we specifically deleted TMEM63B in ISCs by crossing KI mice with *LGR5-Cre* mice (Ctrl, *Tmem63b^HA-fl/HA-f^*^l^; cKO, *LGR5-Cre*; *Tmem63b^HA-fl/HA-f^*^l^). Moreover, we assessed the GI motility by examining the fecal morphology to investigate the function of TMEM63B within the GI tract.

**Figure 2:**
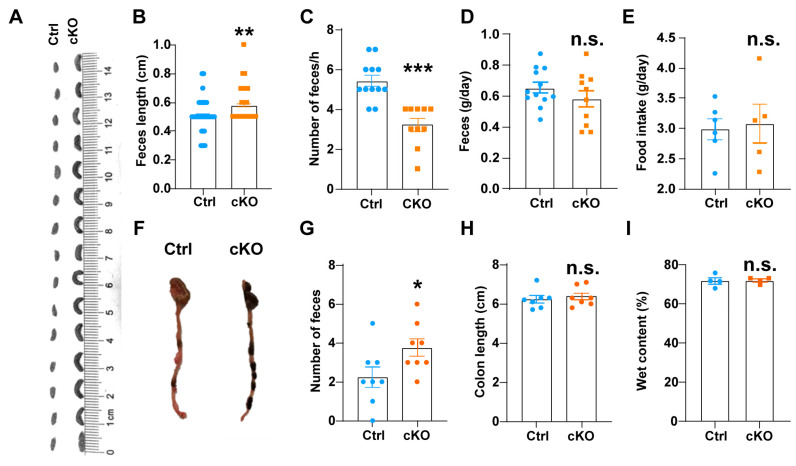
TMEM63B is essential for GI motility in mice. (**A**) Representative images of dried feces collected from control and cKO mice. (**B**) The length of stools expelled per mouse during 1 h of collection from the control (n = 12 mice) and cKO mice (n = 10 mice) (unpaired two-tailed *t* test: ** *p* < 0.01). (**C**) The number of stools expelled per mouse during 1 h of collection from control (n = 12 mice) and cKO mice (n = 10 mice) (unpaired two-tailed *t* test: *** *p* < 0.001). (**D**) The mass of stools expelled per mouse during 1 day of collection from the control (n = 12 mice) and cKO mice (n = 10 mice) (unpaired two-tailed *t* test: n.s., no significance). (**E**) The daily food intake of the control (n = 6 mice) and cKO mice (n = 5 mice) (unpaired two-tailed *t* test: n.s., no significance). (**F**) Representative images of feces retained in the colons of control and cKO mice. (**G**) The number of stools retained in the colons of control and cKO mice (n = 8 mice per group) (unpaired two-tailed *t* test: * *p* < 0.05). (**H**) The colon length of the control and cKO mice (n = 7 mice per group) (unpaired two-tailed *t* test: n.s., no significance). (**I**) Water content present in the stool samples from (**F**) as a percent of the total composition (n = 4 mice per group, unpaired two-tailed *t* test: n.s., no significance).

First, we conducted a comparative analysis of fecal samples from the control and TMEM63B cKO mice under ad libitum feeding conditions. Our results showed a significant increase in fecal length in TMEM63B cKO mice (Figure 2A,B). Furthermore, there was a notable decrease in defecation frequency per hour for cKO mice (Figure 2C), while no significant differences were observed regarding fecal weight or daily food intake between the groups (Figure 2D,E). To further elucidate the impact of TMEM63B deletion on ISC function and GI motility, we examined the retained fecal pellets within the colons of both control and TMEM63B cKO mice. The results showed a significant increase in the number of retained fecal pellets within the colons of cKO mice compared to controls (Figure 2F,G). Notably, no significant differences were observed in the colon length or water weight of the retained feces between the two groups (Figure 2H,I), suggesting that TMEM63B deletion specifically affects small intestine function rather than large intestine function. Collectively, these findings provide evidence that the deletion of TMEM63B in ISCs impairs GI tract motility in mice.

### 3.3. Deletion of TMEM63B in ISCs Reduces Gastrointestinal Digestion

**Figure 3:**
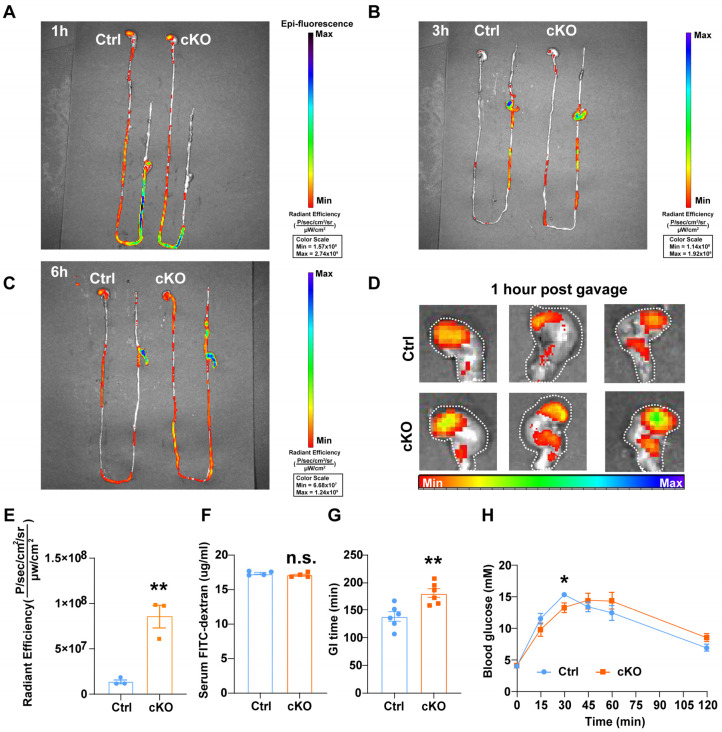
TMEM63B mediates gastric digestion and intestinal transit in mice. (**A**–**C**) Representative images of FITC-dextran distribution at different times in the gastrointestinal tract of control and cKO mice. (**D**) Representative images of the FITC-dextran distribution in the stomachs of control and cKO mice (n = 3 mice per group). (**E**) Quantification of gastric emptying intensity after the administration of FITC-dextran in the control and cKO mice (n = 3 mice per group, unpaired two-tailed *t* test: ** *p* < 0.01). (**F**) Measurement of intestinal permeability by quantifying the serum levels of FITC-dextran from the control and cKO mice (n = 4 mice per group, unpaired two-tailed *t* test: n.s., no significance). (**G**) Quantification of GI transit time measured in control and cKO mice (n = 6 mice per group, unpaired two-tailed *t* test: ** *p* < 0.01). (**H**) Blood glucose levels after gavage sucrose in the control and cKO mice (n = 6 mice per group, unpaired two-tailed *t* test: * *p* < 0.05).

We then assessed the gastrointestinal transit time by examining the distribution of a fluorescent dye administered orally along the gastrointestinal tract. The intensity of fluorescence was used to quantify the amount of dye present. The TMEM63B cKO and control mice were sacrificed at 1 h, 3 h, and 6 h after gavage with FITC-dextran. Whole gastrointestinal tracts were then collected, and fluorescent dye distributions were visualized using IVIS-spectrum imaging. There was a significant retention of fluorescent dye in the stomachs or intestines of TMEM63B cKO mice compared to control mice at different times (Figure 3A–C). Notably, substantial amounts of dye remained in the stomach of TMEM63B cKO mice at 1 h, whereas in control mice, it had already reached the cecum by this time (Figure 3A,D,E). Serum samples collected at 4 h after administering the dye showed no significant difference in the dye concentration between cKO and control mice, indicating that the deletion of TMEM63B in ISCs did not affect intestinal permeability (Figure 3F). Additionally, we evaluated the gastrointestinal digestion and metabolism time by treating the mice with a paste containing carmine red and methylcellulose in specific proportions and measuring the excretion time for the first appearance of red stool. The excretion time for red feces was significantly prolonged in cKO mice (Figure 3G), further supporting impaired digestive function and metabolism due to TMEM63B deletion. To evaluate the nutrient absorption and digestion efficiency, both control and cKO mice were orally administered with sucrose, a disaccharide that requires intestinal digestion to be converted into monosaccharides. Next, the blood glucose levels were measured. Our results showed that blood glucose levels peaked at 30 min followed by a rapid decline in control mice (Figure 3H). In contrast, the TMEM63B cKO mice exhibited a slower rise to peak blood glucose levels which sustained up to 60 min before declining (Figure 3H). Overall, these results suggest that the deletion of TMEM63B in ISCs leads to the functional alterations in the intestines through affecting nutrient digestion and metabolism.

### 3.4. TMEM63B Deficiency Disrupts ISCs Proliferation

**Figure 4:**
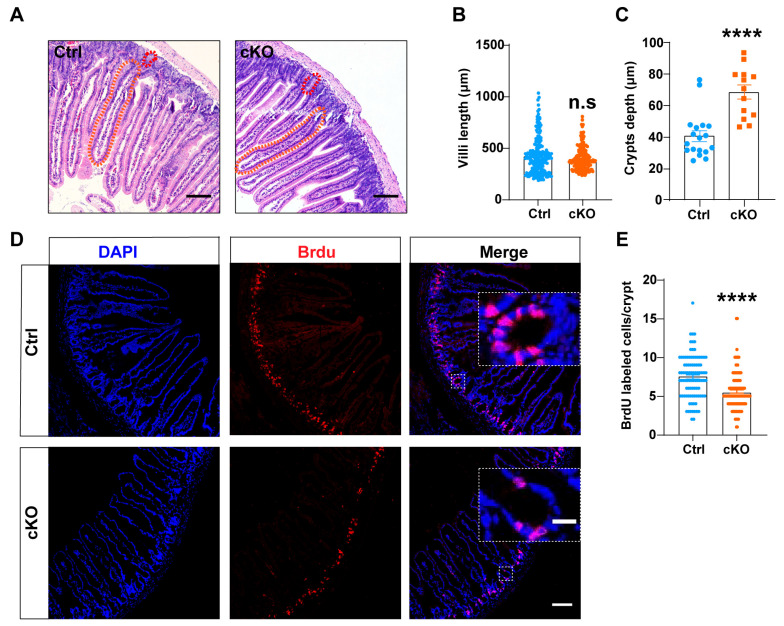
Deletion of TMEM63B resulted in reduced proliferation of ISCs. (**A**) Representative images of H&E staining for villi (orange circle) and crypts (red circle) in control and cKO mice. Scale bars: 100 μm. (**B**) Quantification of villi length in the control and cKO mice (n = 3 mice per group, unpaired two-tailed *t* test: n.s., no significance). (**C**) Quantification of crypts depth in the control and cKO mice (n = 3 mice per group, unpaired two-tailed *t* test: **** *p* < 0.0001). (**D**) Representative images of immunohistochemistry staining using jejunum sections from control and cKO mice after 1 h of BrdU intraperitoneal administration. Nuclei were stained with DAPI (blue), while BrdU-positive nuclei are shown in red. The large dotted frames represent an enlargement of the small dotted frames. The scale bars represent 100 μm for low-magnification images (small dotted frame) and 20 μm for high-magnification images (large dotted frame). (**E**) Quantification of BrdU-positive cells in each crypt of control and cKO mice (n = 3 mice per group, unpaired two-tailed *t* test: **** *p* < 0.0001).

To determine the mechanisms underlying the TMEM63B deletion-induced deceleration of gastrointestinal motility and the impairment of gastrointestinal digestion, we first employed H&E staining to investigate the morphology of small intestinal villi and crypts in the jejunum. Interestingly, we observed a modest decrease in the overall length of cKO villi (Figure 4A,B), while there was a more substantial increase in the crypts depth of cKO mice (Figure 4A,C). Moreover, the decreased ratio of villi height to crypts depth (V/C), which represents absorption capacity, further elucidates impaired digestion within the small intestine of cKO mice. To evaluate epithelial cell proliferation within the small intestine, BrdU was administered as an indicator for S-phase cell proliferation in 8-week-old mice. Notably, BrdU-positive cells in jejunal crypts were significantly reduced after 1 h of BrdU administration in cKO mice (Figure 4D,E). Together, these data suggest that the deletion of TMEM63B leads to a reduction in stem cell proliferation originating from the crypts, which is likely responsible for both prolonged crypt development and impaired digestion.

### 3.5. TMEM63B Deficiency Exacerbates Colitis

**Figure 5:**
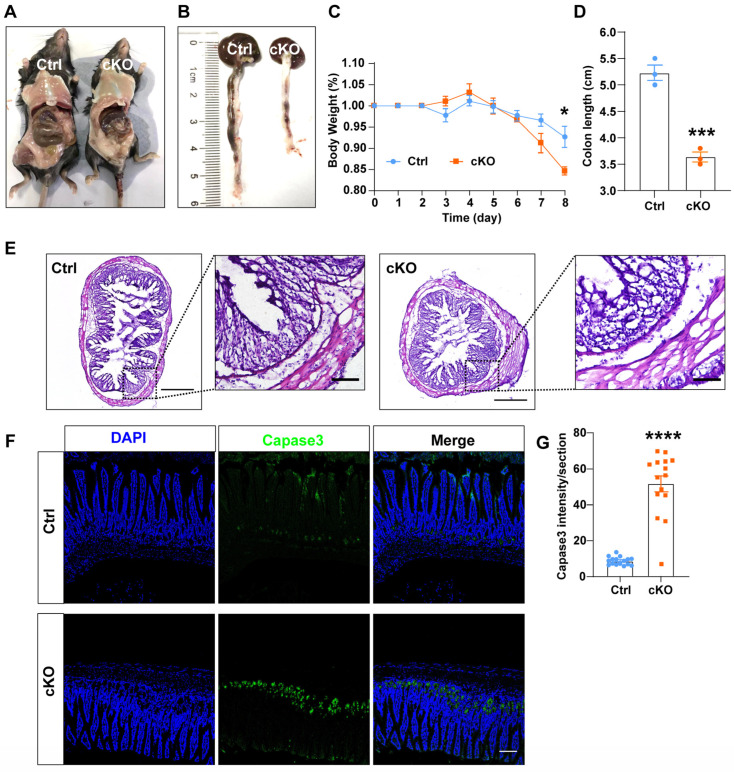
TMEM63B deficiency exacerbates the development of colitis in mice induced by DSS treatment. (**A**) Representative images depict control and cKO mice treated with DSS on day 8. (**B**) Representative colon images from the control and cKO mice are as shown in (**A**). (**C**) Body weight changes expressed as a percentage of initial weight for control and cKO mice following DSS treatment (n = 3 mice per group, unpaired two-tailed *t* test: * *p* < 0.05). (**D**) Quantification of the effect of DSS treatment on colon length in control and cKO mice (n = 3 mice per group, unpaired two-tailed *t* test: *** *p* < 0.001). (**E**) Representative images for H&E staining of colon sections from the control and cKO mice at indicated time points after DSS treatment. The scale bars represent 200 μm for low-magnification images and 100 μm for high-magnification images. (**F**) Representative images for immunofluorescence staining using jejunum samples of control and cKO mice (Blue: nuclei; Green: Capase3, scale bar = 100 µm). (**G**) Quantification of the intensity of caspase3 in jejunum crypts of control and cKO mice after DSS treatment (n = 3 mice per group, unpaired two-tailed *t* test: **** *p* < 0.0001).

The intestinal epithelium serves as a crucial barrier to maintain intestinal health, and compromised function can lead to enteritis. ISCs continuously proliferate to generate new intestinal epithelial cells for tissue repair. However, the insufficient proliferation of these cells can exacerbate enteritis. To assess the impact of TMEM63B on ISC proliferation, we treated mice with DSS (a colitis model) and found that TMEM63B cKO mice exhibited more severe colitis symptoms compared to controls (Figure 5A,B), including significant body weight loss and shorter colon length (Figure 5A–D). Histopathological analysis showed the extensive infiltration of inflammatory cells in both groups, but cKO mice showed an increased presence of liquefied vacuoles within the epithelium indicating substantial damage (Figure 5E). Additionally, the immunofluorescence results showed higher levels of apoptosis in TMEM63B cKO mice compared to the controls (Figure 5F,G). In summary, these findings suggest that the deletion of TMEM63B in ISCs impairs the functionality of intestinal epithelial cells by affecting the proliferation of ISCs.

## 4. Discussion

Since the 18th century, the crucial role of intestinal motility and digestion has been widely acknowledged [37]. Various factors, including the central nervous system (CNS), enteric nervous system (ENS), intestinal cells, and certain immune mediators, regulate intestinal motility and digestion [38,39]. Among these factors, intestinal endocrine cells and epithelial cells are derived from the proliferation and differentiation of ISCs. In recent years, there has been increasing attention paid to the physical property changes of cytoplasm in ISCs [40,41,42,43]. When food enters the intestines, digestion can alter the intestinal microenvironment [18], which activates mechanosensitive ion channels that are extensively expressed in the intestines. Recent studies demonstrated that TrpA1 and PIEZO1, which are abundantly expressed on ISCs in *Drosophila*, can be activated by mechanical stimulation to induce calcium ion influx and ultimately regulate ISC proliferation [19,36]. Similarly, Tallapragada et al. showed that mechanical force-induced calcium influx mediated by PIEZO1 leads to the formation of more stem cell zones (SCZs) in cultured intestinal organoids [21]. Furthermore, when these organoids were incubated with GdCl_3_ (a calcium channel blocker), both calcium influx and SCZ formation induced by mechanical activation were significantly reduced. These findings collectively demonstrate that the proliferation of ISCs is facilitated by calcium influx mediated through mechanosensitive ion channels. Consequently, these intriguing findings prompt us to identify previously undiscovered mechanosensitive ion channels that are expressed in the intestines. Importantly, this study identified TMEM63B as a novel mechanosensitive ion channel abundantly expressed in ISCs that regulate both intestinal motility and digestion.

The mechanical patterning plays a crucial role in the formation of intestinal crypts, where compressive stresses within the epithelial monolayer drive buckling and establish characteristic tissue curvature and length scales [44,45,46,47,48,49,50]. This process facilitates the development of crypts that are essential for cell proliferation and renewal in the intestines. In this study, we confirmed that TMEM63B is highly expressed in ISCs. When comparing control and *Tmem63b* cKO mice provided with ad libitum food access, significant changes were observed in the fecal patterns of cKO mice, including a notable increase in feces length and a significantly higher number of retained fecal pellets within the colon. Whole gastrointestinal transit measurements using red-dye-infused food revealed a significantly prolonged excretion time for the first red-colored feces in cKO mice. In vivo imaging experiments utilizing FITC-dextran administration demonstrated an increased retention of fluorescent dye within the stomachs of cKO mice, further indicating impaired peristalsis. Additionally, when sucrose was administered via gavage to metabolize into glucose for absorption, blood glucose concentrations were measured at different time points. The results showed a prolonged time to reach peak blood glucose levels in cKO mice, suggesting weakened digestive and absorptive capabilities. Collectively, these findings underscore the crucial role of TMEM63B in regulating intestinal motility.

In the colitis model, the TMEM63B deletion in ISC mice exhibited a more severe intestinal injury, indicating that the deletion of TMEM63B impaired the reparative capacity of intestinal epithelial cells. We investigated whether this weakened ability to restore damaged epithelial cells was attributed to the impaired proliferation of ISCs. Therefore, we employed BrdU labeling to assess the proliferation rate of ISCs. Interestingly, the TMEM63B cKO mice displayed a significant reduction in cell numbers per crypt. These findings suggest that the deletion of TMEM63B, as a mechanosensitive ion channel, its deletion induces a decline in ISC proliferation, thereby contributing to compromised intestinal function. In conclusion, this study enhances our comprehension of mechanosensitive ion channels in the small intestine and provides potential therapeutic targets for enteritis treatment.

## 5. Conclusions

In this study, we identified TMEM63B as a mechanosensitive ion channel that is highly expressed in ISCs. TMEM63B played a crucial role in mediating the proliferation of ISCs, thereby facilitating intestinal self-renewal. The deletion of TMEM63B in ISCs resulted in impaired ISC proliferation and compromised intestinal function.

## Data Availability

The data presented in this study are available from the corresponding author upon reasonable request.
